# Social Networking in Health System for Knowledge Translation

**DOI:** 10.5681/jcvtr.2014.015

**Published:** 2014-03-21

**Authors:** Hafez Mohammadhassanzadeh, Farhad Shokraneh

**Affiliations:** ^1^Research Center for Pharmaceutical Nanotechnology, Tabriz University of Medical Sciences, Tabriz, Iran; ^2^Cochrane Schizophrenia Group, the Institute of Mental Health, a partnership between the University of Nottingham and Nottinghamshire Healthcare NHS Trust, UK

Interaction among health system sections is one of the main contributing factors for progress of biomedical fields. Such interaction requires sharing the knowledge in properly designed networks of researchers and clinicians where experience meets expertise to create new knowledge. As an infrastructure for approaching this idea, there is a need for development of integrated databases for identification, classification, and discovery of the knowledge about researchers and clinicians to analyze their links in a network. In this case, Social Network Analysis (SNA) works as a society-oriented prototype to explore the structural patterns of social connections;^1^ hence it renders both visual and statistical representations of interpersonal relations.^2^ SNA, a subset of ‘sociometry’ coined by J. L. Moreno^3^, presents the methodology to analyze social relations. It is composed of nodes (individuals), the smallest unit in a network, and lines (relations), a tie between two nodes. Likewise, ego-networks are kinds of social networks that focus on certain nodes and are called egos.^1^ They also propose highly remarkable information in order to comprehend the structure and nature of relationships, and interaction within a scientific community.^1^

Here, we propose the development of a database benefiting SNA to get the required inputs for a social interface involving various groups of health system. Integration of health system groups by means of new methodologies and technologies could provide valuable outputs for knowledge flow among these groups. SNA has also been suggested as a pragmatic methodology to delineate interaction models in healthcare settings.^4-8^ Furthermore, we aim to present a proposal to integrate the health system groups through the SNA methodology as well as social media technology.

## Inputs

### Phase I

### 1. Clinical practice step

1.1. Importing data such as the clinicians’ names, their specialty, their practical efforts, and so on in a clinical information system to register, analyze, and report the practical outcome of each clinician;

1.2. Designing a database for medical specialists;

1.3. Linking between the clinical information system and the database of medical specialties for obtaining the outputs such as rank of clinicians in any specialty and filing as well as tracing the successful ones;

1.4. Analyzing clinicians’ interpersonal relations with other clinicians, their colleagues, team members, and consultants through the ego-networks which lead in detection of core clinicians and clinical teams.

It should be stated that ego-network is one of the new approaches we are exploring in order to develop new tools that will help policy/decision-makers and academia to jointly plan, implement, monitor, and evaluate investments in the network.

**Figure 1:**
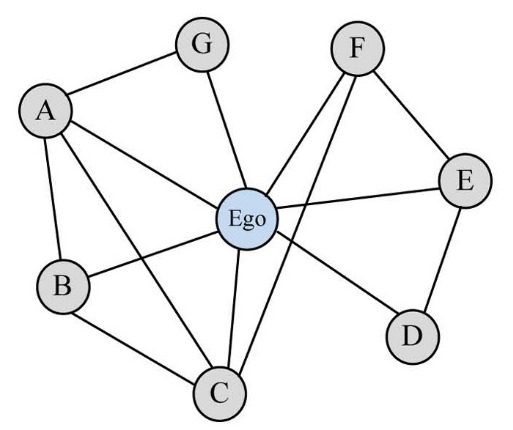
Structure of a simple ego-network

In Figure 1, as an example of ego-network, ego node is connected to all nodes showing all relations of an individual. Ego-networks detect relations as power points, ‘structure holes’ or scientific gaps, as well as possible weak points, and help in forming multidisciplinary fields and ‘unified scientific community’. Nodes A and G are linked through a line, whereas there is no line between nodes D and C which is called a ‘structure hole’. As density of the network decreases, more ‘structural holes’ are likely to open in the ‘social fabric’. These holes and how/where they are distributed can be a source of inequality (in both the strict mathematical sense and the sociological sense) among actors embedded in networks.

### 2. Research step

2.1. Seeking papers indexed in citation-based scientific resources such as Scopus, Thomson Reuters’ citation indexes, and Google Scholar based on research fields;

2.2. Identifying top researchers of each field based on ‘citation’ indicator;

2.3. Analyzing papers of the researchers using the ego-network;

2.4. Specifying core research teams in each field via the ego-network.

### 3. Link step

3.1. Connecting core clinical teams to core research groups through the SNA methodology.

### Phase II

1. Development of a multi-purpose social media platform for the health system groups;

2. Definition of access levels for each kind of users.

## Outputs

**Figure 2:**
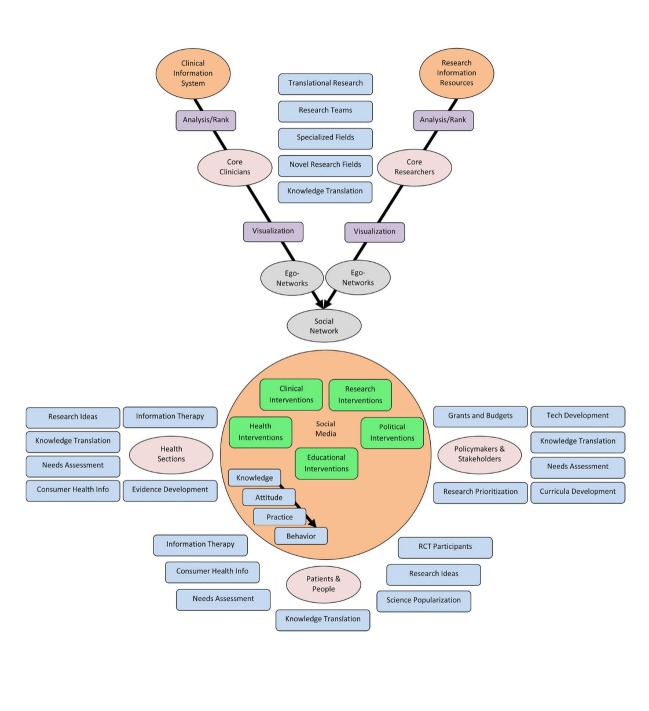
Proposed integration among health system groups; orange areas show technologic systems as information and knowledge production and presentation media; violet parts present analyses through software programs; pink areas demonstrate health system groups; grey sections present networks as outputs of software programs; blue boxes are added values of integration for different health system groups; and green sections are categorized interventions which happen within the social media.

Bridging the gaps among health system groups through a social media could have various added values for health system. Presented proposal could make research, educational, clinical, health, and political interventions possible in an integrated environment (Figure 2).

### Researchers’ and clinicians’ interactions

Researchers and clinicians could find each other through SNA-based networks within social media; therefore, clinical and non-clinical researchers could integrate their interests to work on translational studies, at the same time they are both connected to related clinicians who may facilitate the selection of novel research topics and have synergic effect on clinical trials and application of findings. Such integration leads to the development of new multidisciplinary research teams and specialized fields contributing research centers, clinical settings, and health sections. On the other hand, both of involved groups will be aware of current progress in clinics and research areas that make translation of practical and scientific knowledge possible among researchers, clinicians, and people. Simultaneously, researchers could discover new ideas through the results of needs assessment by health sections and clinicians could utilize developed high-level evidences like clinical practice guidelines as a part of knowledge translation initiatives.

### Health sections’ interactions

While health sections could develop high quality evidences such as clinical practice guidelines, systematic reviews, and health technology assessments based on novel research findings, they also could provide information therapy and consumer health information initiatives for patients and people respectively based on needs assessment among involved people in the social media. Beside such knowledge translation efforts, health system research and population-based studies could be possible for health researchers presenting novel research ideas.

### Policymakers and stakeholders

Grants and budgets are essential for each health system program and health policymakers and stakeholders need enough evidence to decide on any suggested plan. Regarding provision of knowledge translation requirements for decision makers by social media, they could perform needs assessment for new health programs, research prioritization, new academic curricula, and new technologies development.

### People-patient interactions

Presented social media provide a democratic platform for people as they are able to play an active role in health system by presenting their needs and questions (research ideas), and acquire reliable health information through health sections. These interactions result in the popularization of science that in turn facilitates people-patient participation in research studies such as clinical trials.

### Knowledge flow and changes in system

Optimized social media make health system groups closer to each other, bridging knowledge gaps among them. However, the most important effect of such environment is the influence of knowledge on changing attitude, practice, and behavior of health system groups during the time and directing them towards a knowledge-based health system. It means that health groups will play an active role in their health. Due to the changes happening during time, knowledge interventions gradually could turn the passive, mechanical and short-term programs raising resistance among different health groups into active, evidence-based, long-term programs involving all health system groups. These indicate more acceptability.^9^

In brief, the implementation of this project may assist in the progression in health, science, and technology, through improvement in knowledge translation among health community. Finally, integration of health system utilizing new technologies and methodologies will have additionalvalues for the system beyond what current systems suggest.

## Ethical issues

Not applicable.

## Competing interests

The authors declare that there is no competing interests.
